# Sparse cliques trump scale-free networks in coordination and competition

**DOI:** 10.1038/srep21870

**Published:** 2016-02-22

**Authors:** David A. Gianetto, Babak Heydari

**Affiliations:** 1 School of Systems and Enterprises, Stevens Institute of Technology, Hoboken NJ, USA; 2 Raytheon Space and Airborne Systems, El Segundo CA, USA

## Abstract

Cooperative behavior, a natural, pervasive and yet puzzling phenomenon, can be significantly enhanced by networks. Many studies have shown how global network characteristics affect cooperation; however, it is difficult to understand how this occurs based on global factors alone, low-level network building blocks, or *motifs* are necessary. In this work, we systematically alter the structure of scale-free and clique networks and show, through a stochastic evolutionary game theory model, that cooperation on cliques increases linearly with *community motif* count. We further show that, for reactive stochastic strategies, network modularity improves cooperation in the *anti-coordination* Snowdrift game and the Prisoner’s Dilemma game but not in the Stag Hunt coordination game. We also confirm the negative effect of the scale-free graph on cooperation when effective payoffs are used. On the flip side, clique graphs are highly cooperative across social environments. Adding cycles to the acyclic scale-free graph increases cooperation when multiple games are considered; however, cycles have the opposite effect on how forgiving agents are when playing the Prisoner’s Dilemma game.

The puzzling existence of cooperation in the face of the seemingly selfish process of natural selection has occupied researchers across many disciplines for over 30 years^1^,^2^. This interest extends beyond the most common biological and social contexts^2^,^3^ to a wide array of literature including, the theory of the firm in business where coordination of functions and competition with external entities are key considerations^4^, coordination during preparation for natural disasters in emergency management literature^5^, how cooperation influences morality emergence^6^, in the psychology literature, and the physical mechanisms that give rise to human cooperation provided by neuroscience^7^,^8^, to name a few. A common way to model cooperation is by evolving an interacting population of social dilemma game players; a canonical game is the Prisoner’s Dilemma^9^,^10^.

This line of research has revealed a host of factors that contribute to the survival of cooperation^11^ including, remarkably, the interaction structure itself (see^12^ for a review). Evolving organisms often reproduce locally which in turn causes feedback between locally distributed traits and genetic relatedness between traits, this leads to interaction assortment and *population viscosity*^13^,^14^ that sustains cooperative behavior; however, what properties of structure drive what characteristics of cooperation and how? One way to tackle this problem that has proven useful is to consider evolving players of social dilemma games within a network, where the games provide a framework for understanding the simplest local interactions and the network frames community-level and global interaction^12^. This general approach has shown that relatively sparse connectivity^15^ or less frequent interaction^16^ helps encourage cooperation. More modular structures also encourages cooperation relative to networks with less separable communities^17^. It has also been commonly shown that heterogeneous networks, such as scale-free graphs with a power-law degree distribution dependence^18^, encourage cooperation^18^,^19^,^20^. Even so, it turns out that the advantage of cooperators disappears in scale-free networks when payoffs are averaged over neighbors rather than summed, because hubs are easily invaded by defectors even if cooperators favor hub locations^21^,^22^. Cycle length within a network is also a key characteristic and the strongest evolutionary dynamics tend to occur within cycles^23^. Short and long cycle effects compete with one another; short cycles pull cooperation lower while long cycles drive cooperation higher in the Prisoner’s Dilemma game^24^ and Ref. 25 shows that short cycles similarly limit cooperation.

In the present investigation into how the properties of structure drive cooperation we consider cooperation over four two-person, symmetric, stylized games common in the literature (i.e., Stag Hunt (SH), Snowdrift (SD), Harmony Game (HG), and Prisoner’s Dilemma (PD)), rather than compare the effects of one or two games, which is most common. In a social context any individual game may prove relevant for a given situation at a certain point in time and so the representative game is subject to change over time^26^,^27^; however, altering network structure to adapt to changing circumstances is costly^28^. In fact, network structure has been shown to actually inhibit cooperation when costs are high relative to benefits in the Snowdrift game^29^ and the common assumption of heterogeneous degree distribution encouraging cooperation is negated by participation costs^30^ and payoff averaging^21^,^22^. Therefore, in order to design enduring structures that influence behaviors either towards or away from cooperation we need to consider a wide range of social situations that the population may encounter.

How networks affect cooperation is usually considered as relative to average global structural properties, but this approach cannot give an accurate picture of local network characteristics nor their influence on evolving processes or functions, this is the purview of network *motifs*^31^. Motifs are common, repeating, statistically significant, subgraph building blocks, that lie below both the global and community level^32^,^33^,^34^ and just above the lowest node and link level of a network. The notion of network motifs applies to biological, social, and information network contexts; where, for example, motifs found in the world wide web are distinct from genetic networks^35^. Motifs, which can be efficiently detected in real-world networks^36^,^37^, are functionally separable *modules* which interact dynamically within an organism and between an organism and its environment to both provide core functionality and adapt to the environment^38^. Since the environment is not static, but changes over time, evolutionary *goals* also vary, therefore, modular structure and network motifs must emerge to improve survival in this dynamic environment^39^. Certain motifs may be indicators of social status in networks^40^ and influence network dynamics as well^41^. Finally, if a range, or *spectrum* of motifs is considered a network hierarchy may be determined, when the network is directed^42^.

In the present work we use network motifs to help us understand the consequences of subtle changes in network structure which have very little effect on average global network characteristics but influence cooperation emergence.

Here forward, we introduce the evolutionary model we have developed for this work, highlight the effect of varying structures on cooperation across multiple games and delve into two example structures which show the lowest average cooperation across games (a scale-free graph) and highest average cooperation across games (a clique-type graph), relative to the fully-connected case. Our results show that networks which lack either sufficient cyclic structures or contain densely connected cliques suffer significantly reduced cooperation, particularly in coordination situations, adding cycles helps here but when the social environment becomes more competitive this can be counterproductive. On the flip side, connecting highly cooperative clique communities together improves cooperation linearly with the frequency of local *community motif* structures, which extend interfaces to neighboring communities and give a cohesive structure for coordination and cooperation locally. Cycles can either help (coordination) or hinder (competition) depending on the social environment that is encouraged by a particular network which spread prevailing community behavioral norms to other communities.

## Results

### Model

The model we have developed for this work operates on four levels: pair-wise games, strategy space, the learning model, and the interaction structure or network. Game play occurs between agents who play two-player repeated games with each of their nearest neighbors and receive payoffs according to the moves of each player, commensurate with the payoff structure of the game, equation (4). We consider four games, including the Prisoner’s Dilemma, or PD (the strongest social dilemma), Stag Hunt (SH), Snow Drift (SD), and the Harmony Game, or HG (the weakest dilemma). During game play, agents, with memory of their opponent’s last move within a repeated game, determine their next move according to their stochastic *reactive* strategy, which is based on the three-component, Markovian, mixed-strategy framework from^43^,^44^, where *y* is the probability to trust on the first move, *p* is the probability to reciprocate a cooperative move and *q* is the probability to forgive defection. Since *y* acts in a single round within a repeated game and the other strategies repeat throughout *y* is plagued by noise, indeed it can be problematic to collect enough samples to show the meaningful evolution of *y* (trust)^17^. Therefore, we treat *y* as a discrete rather than a continuous variable in this work; this treatment decreases *y* noise considerably and improves run convergence. Learning, or strategy updating, is done asynchronously via the Fermi rule, equation (1), from relatively weak to strong selection, 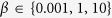. We also employ a social interpretation (which we refer to as **SOC**, see equation (2)) of the Fermi rule where *β* is inversely proportional to the payoff noise across each agent’s nearest neighbors; we describe the model in greater detail in the Methods section.

**Figure 1:**
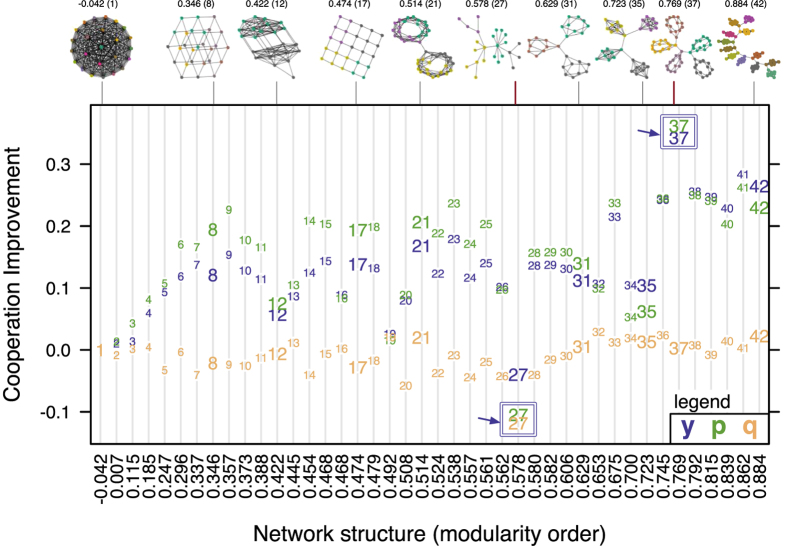
Cooperation change with structure. The figure shows difference in average cooperation 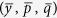 across a set of the four most common two player symmetric games (i.e., Harmony Game, Stag Hunt, Snow Drift, and Prisoner’s Dilemma) between a set of networks in modularity order. The point symbol is the corresponding graph index from the networks figure in the Methods section. The network modularity (*Q* in^32^) is shown along the horizontal axis; sample graphs are shown above the plot. Graph node color represents community membership, which we determined using the random-walk method described by^60^. The most significant outliers are the Barabási-Albert (BA) graph^47^,^48^ (graph index 27) which experiences the least improvement (negative) in cooperation and the clique graph (graph index 37) which evolves much higher cooperation (both compared to the full graph). Both of these cases are noted in the figure with a blue arrow.

In our quest to increase the model’s sensitivity and performance we abandoned the synchronous updating rule, replicator equation, and stochastic *y* methods from prior work^17^ in favor of asynchronous updating, the Fermi rule, and a discrete *y* variable. These changes also allowed us to verify cooperation improvement (shown in Fig. 1) with modularity, studied by^17^, under different updating and evolutionary rules. In addition, we tested more modular structures up to a maximum modularity *Q*^32^ of nearly 0.9. A graph generator was required to test the modularity range from 0.6 to 0.9 in nearly equal increments; we developed the generator based on^45^.

For payoffs, we use the average performance of a node over its neighborhood (see equation (3)) rather than a the neighborhood sum used by^46^. We chose this approach both to associate results of this study with prior modularity work^17^ and to increase the generality of our results since the averaging rule produces dynamics invariant to payoff matrix translations^22^. Averaging payoffs may negate the benefit of the scale-free network^21^ but sparse cliques still enjoy broad cooperation improvement, which we shall discuss in the next section.

### Cooperation and modularity

**Figure 2:**
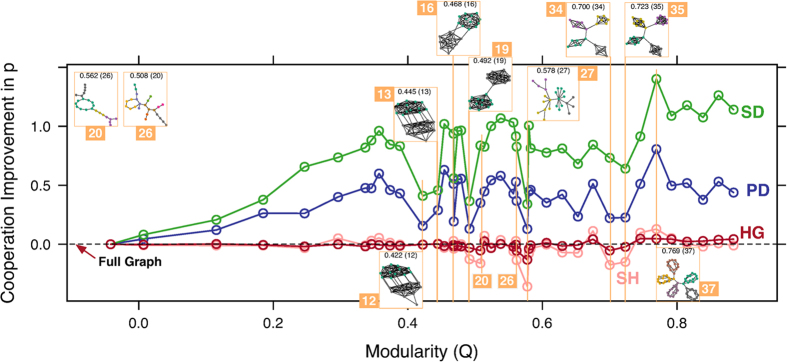
Effect of modularity on cooperation. The figure shows Cooperation *p* (reciprocity) as relative to Modularity (*Q*) conditioned by game, one curve for each game. Significant cooperation changes (e.g., dropouts) are indicated by vertical lines with the corresponding network attached, along with the corresponding graph index from the network figure in the Methods section. Index 12, 13, 16, 19, 27, 34 and 35 correspond to local cooperation depressions (dropouts), network 37 from Fig. 1 is also shown. Approximately acyclic graphs are highlighted on the graph (20, 26) for each game; networks are shown for each on the plot.

Broadly considering the results in Fig. 1, the network with the lowest overall change in cooperation relative to the full graph, across all games, is the Barabási-Albert (BA) graph^47^,^48^ in Fig. 1 (graph index 27). Cooperation change relative to each game is shown in Fig. 2 where significant drops in cooperation occur along the spectrum from low to high modularity. The most prominent dropouts in Fig. 2 fall into three categories, those with highly connected cliques (index 12, 13, 16, 19, 34, 35 in Fig. 2) whose modules approximate a full graph and so are less cooperative, the BA graph (index 27 in Fig. 2) which has reduced cooperation due to frequent invasion of hubs by defectors^21^,^22^, and approximately acyclic graphs (index 20, 26 in Fig. 2). The dropouts are the largest in the BA graph, especially in the Snowdrift and Stag Hunt games, loss of cooperation in the later is significant enough to reduce average cooperation across all games below the full graph (Fig. 1).

**Figure 3:**
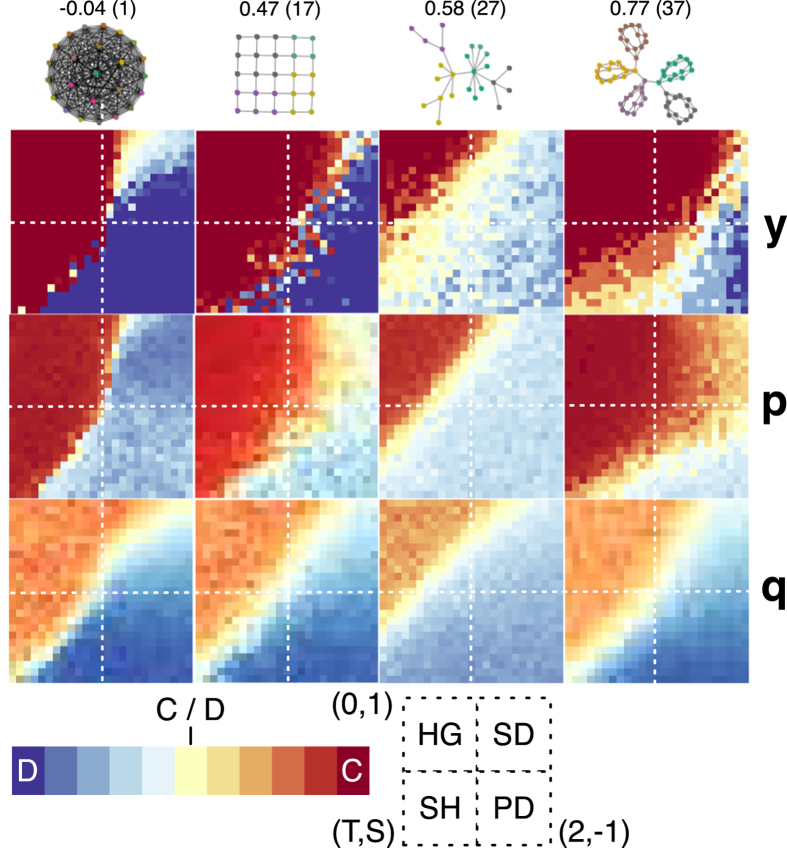
ST Plane versus structure. The figure shows cooperation across the ST-plane for y (top row), p, and q (bottom row) for the full graph (left column), the BA graph (index 27), the 5 × 5 square lattice (index 17), and the clique graph (right column, index 37). Coordinates within each ST-plane represent the payoffs of the Harmony Game (HG), Snowdrift (SD), Stag Hunt (SH), and Prisoner’s Dilemma (PD) games; divisions between each game are shown as white dashed lines and the game region is noted in the bottom of the figure. See SI1 for detailed plots comparing each network with the the full graph by game.

From the ST-plane results in Fig. 3, forgiveness is affected the least by changes in network structure. The simple 5 × 5 lattice structure (see SI1 for lattice details) improves cooperation significantly across the plane but the Snowdrift game shows the most improvement, but cooperation dominates the majority of the ST plane for the clique graph (rightmost column in Fig. 3). Since payoffs are averaged based on a node’s performance against each of its neighbors (confirming^21^,^22^ for reactive stochastic strategies), cooperation in the BA graph (27 in Fig. 3) is reduced across all games, even compared with the fully connected graph (leftmost column in Fig. 3).

Beyond the payoff structure effect reported by^21^,^22^, we hypothesize that some of the cooperation erosion in the BA graph is due to its *acyclic* nature rather than its preferential attachment property or heterogeneous degree distribution per se. The evolutionary dynamics of games on cycles can favor cooperation, especially for more competitive games like the Prisoner’s Dilemma^23^, and analytical work has shown that a combination of relatively large girth and fewer short cycles should improve cooperation^24^, so an undefined girth (acyclic) may limit cooperation likewise. To test this, we developed an experiment in the next section that attempts to increase scale-free graph competition through adding cycles from single-edge changes to the network.

### Scale-free graph edge experiment

**Figure 4:**
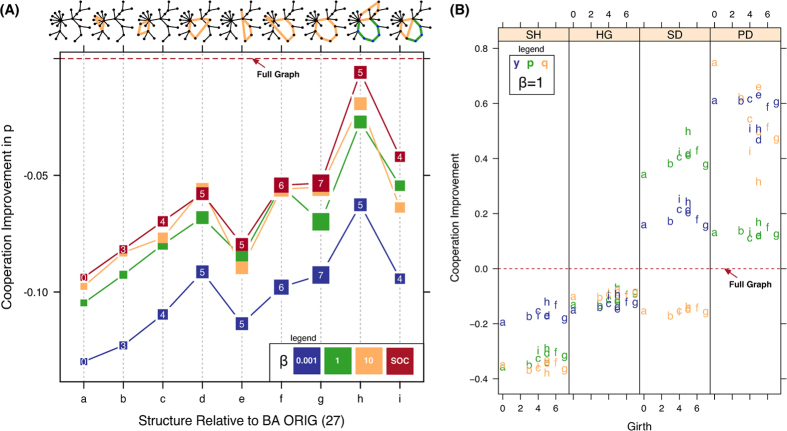
Effect of cycles on cooperation. Panel (**A**) shows the change in reciprocity *p* across all games by single-edge additions to the BA graph (Fig. 1 index 27). Each point is represented by a square with length (width) proportional to the *girth* of the graph, or the length of the shortest cycle. The girth value is shown within each data point square for the 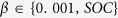. The graph corresponding to each data point is shown above; the shortest cycle is shown in orange. The vertical axis represents the average reciprocal cooperation (*p*) level across games (PD, SD, HG, SH) relative to the full graph (Fig. 1 index 1). The four selection strength *β* curves are shown (0.001, 1, 10 and the **SOC** adaptive method, equation (2)). Panel (**B**) conditions the cooperation results by game relative to the full graph, for 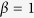. The full graph is indicated by the horizontal red line. Point symbols are for each respective point index in panel (**A**). A summary of the effects in panel (**B**) is shown in Table 1.

To provide evidence for the effect of cycles on cooperation we constructed a simple illustrative example by systematically adding cycles of different lengths to the BA graph, thus gradually increasing its girth. From Fig. 4(A), when girth rises average cooperation across all games increases proportionally. However, girth fails to tell the complete story. If higher degree hubs are included in the cycle (Fig. 4(A) point d), cooperation is higher than the same length cycle that connects through a lower degree node (Fig. 4(A) point e). Cooperation does not continually improve as cycle length increases, a limit is reached where new cycles must be added to achieve further improvement. If we add another cycle (Fig. 4(A) point h) cooperation improves but if a new cycle is added by splitting a large cycle (Fig. 4(A) point i), thus decreasing girth, cooperation decreases relative to the two-cycle case (point h). These effects are similar in nature from strong to weak selection, including the **SOC** *β* rule, equation (2).

Since our model separates behaviors into three factors, trust (*y*), reciprocity (*p*), and forgiveness (*q*), we can see some of the inner workings of the effect cycles have on cooperation, shown in Fig. 4(B). First we see that in all respects BA-graph cooperation in the PD game is higher than the full graph; however, the coordination games (SH and HG) lose ground and are less cooperative and the anti-coordination game, SD, decreases in cooperation likewise but is able to remain slightly more cooperative than the full graph due to its modularity. The dearth of cycles does also tend to make cooperation more difficult in coordination situations, though the effect is modest. Moreover, there is a marked difference between games; the positive effect of girth on *y* and *p* strengthens in the SH and SD games but then reverses in the PD game where *y* and *q* reduce with girth, see Table 1 for a summary of these effects. Long cycles help trust and reciprocity evolve in the anti-coordination game (Snowdrift) but when the game shifts to a more competitive situation (PD) longer cycles actually reduce trust and forgiveness; reciprocity is unaffected. The difference lies in the evolutionary dynamics where long cycles provide more opportunities to dampen defection through forgiveness than short cycles. Further, when a cycle is lengthened a higher proportion of nodes in the network are within the cycle, which are known to favor cooperators over defectors due to the supporting effect of cooperators just behind the boundary between communities; defectors fail to support in kind^23^. In the more competitive PD game the ability of forgivers in the cycle to support boundary nodes that experience defection is compromised, this causes the strategy boundary to move in favor of defectors as cycles lengthen.

Cycles do seem to play a key role in cooperation emergence; however, acheiving highly cooperative structures through adding cycles is difficult, in-fact, doing so by splitting cycles is an ineffective way to increase cooperation. Even though cooperation increases by adding cycles to the BA graph we were not able to meet the cooperation levels of the full graph in our eight edge addition experiment though the social (**SOC**) rule came close to achieving parity (Fig. 4 point h). To create the most cooperative structures, we must start with more modular structures that are initially much more cooperative than the full graph, demonstrated by^17^ for the Prisoner’s Dilemma game.

### Clique graph edge experiment

**Figure 5:**
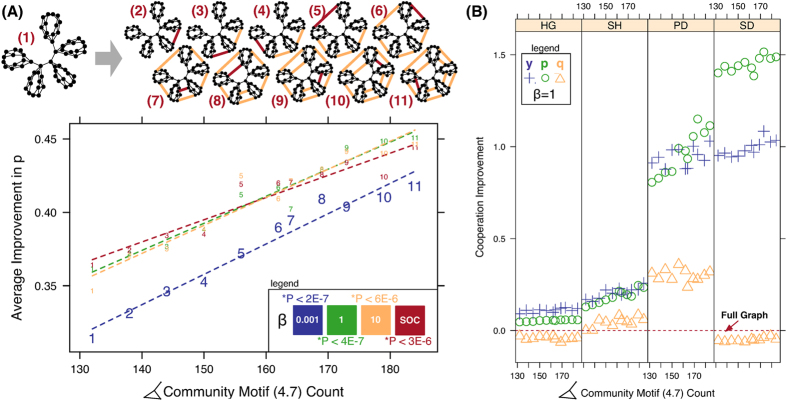
Motif effect on cooperation. Panel (**A**) shows the average change in reciprocity *p* across all games by single-edge additions to the cooperation outlier graph shown in Fig. 1 (graph index 37). The horizontal axis is the quantity of type *community* motifs (i.e., triangle with an extended link). One edge is added (shown in red) between adjacent cliques until all cliques are joined to their neighbors. The network for each point in the plot is shown by an index that corresponds to the point symbol on the plot. Cooperation increases as edges are added to the clique and motif counts increase. A linear model is shown for four *β* cases; a table of linear model P-values is shown on the plot, in all cases the *F-test* two-sided p-value is less than 3*E*-6. Across selection strength cooperation improves by 1% relative to the full graph for each edge added (10% improvement after adding 10 edges). Panel (**B**) consists of four sub-panels that condition the cooperation results by game relative to the full graph, for 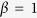. The horizontal axis for each sup-panel is the quantity of *community* motifs. The full graph is indicated by the horizontal red line. A summary of the effects in panel (**B**) is shown in Table 1.

**Figure 6:**
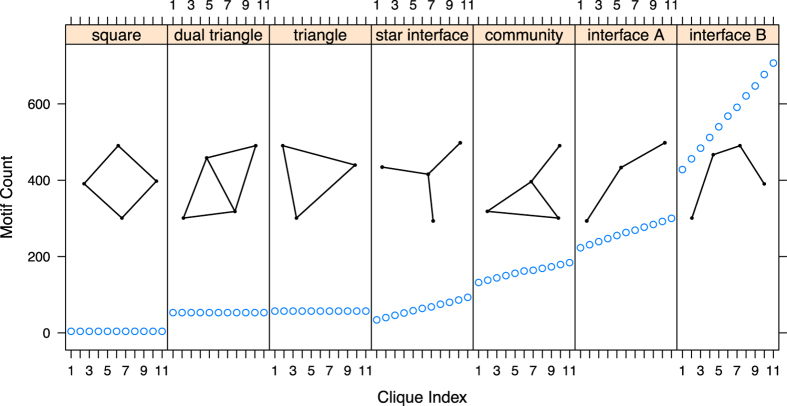
Motif changes between cliques. The figure shows each of the graph *motifs*^35^ of size 3 and 4 that are found within the graphs. In our clique edge-addition experiment we added edges in a way that did not increase short cycles and this is demonstrated by unchanging quantities of *triangle*, *square*, and *dual triangle* motifs. The largest changing motif with a cycle is the *community motif*. Long interface motifs increase linearly throughout the experiment (*interface B* motif) and at the highest rate that indicates an increase in long cycles.

To synthesize a more cooperative environment we start with a network that has the highest average payoff across games (a clique graph in Fig. 1, index 37) and add edges that join communities such that short cycles (e.g., triangular motifs) are not affected. From Fig. 5, average cooperation increases as edges are added. We continue to add edges to create another complete cycle between cliques and cooperation increases likewise. Because the motif changes are controlled only one cyclic motif is affected by these edge additions, namely the *community motif* in Fig. 6, all other changing motifs are acyclic. Since we expect acyclic structures to be less cooperative (Fig. 2) and adding edges between communities *decreases* modularity (9% total decrease for index 11 versus index 1 in Fig. 5(A)) it follows that the community motif is largely responsible for the cooperation improvement in this clique experiment in spite of the opposing effect from lower modularity; in this example cooperation improved by 1% relative to the full graph for each edge added. We found this effect to be robust to selection strength as well as the social (**SOC**) selection rule and confirmed the magnitude of the effect with the replicator equation utilized in^17^.

The motif results are separated by game and *y*, *p*, *q* component in Fig.5(B). There is a considerable difference in the SD (strong *T*) versus SH (weak *T*) games; forgiveness does not evolve in the former (strong) case versus the later (weak) and HG remains unaffected by the *community motif* increase. In contrast with the girth study (Fig. 4), *p* increases with the community motif in all games except HG and no reversal occurs in any game, compared to the PD reversal (Fig. 4) when cycles are added to the BA graph. Since the clique graph provides a highly cooperative environment defection is largely contained, demonstrated by the unchanging forgiveness (*q*) as interfaces with other communities are added.

## Discussion

To summarize, there are four main structural factors that affect cooperation in these settings, some positive and others negative, each act at either the local or global level of the network (see Table 1). These structural factors include connection degree^15^, modularity^17^, and as we have shown in this work, cycles^23^,^24^,^25^ and community motifs.

There are differences, however, in how networks affect cooperation between pure strategy evolution and stochastic strategy evolution. For pure strategies in the PD game^25^, showed that triangles limit cooperation under high noise conditions, but we show that even a single triangle can increase average cooperation across games when stochastic strategies are used across noise levels. This results in a different ideally-cooperative structure for stochastic reactive strategies across games, many long cycles are desired. This contrasts with pure strategies in the PD, where acyclic networks are desired under high noise conditions and randomly connected triangular structures are desired under low noise conditions^25^.

Further, we do confirm the result from^25^, that even a single triangle *decreases* cooperation in the PD game, but limit this effect to the forgiveness *q* part of cooperation (see Fig. 4(B), PD panel, point *b*).

Modularity may be essential for improving cooperation globally^17^ but the modules themselves must be connected sparsely (see Fig. 2), as^15^ suggests with the *b*/*c* > *k* rule, to sustain cooperation. Indeed some of the most modular structures we tested, including graph index 42 in Fig. 1, were significantly less cooperative than the structures in our clique experiment (Fig. 5) due to dense connections within their modules, and cooperation dropouts (i.e., low cooperation though the network is modular) occurred commonly in networks with highly connected modules (see graphs 12, 13, 16, 19, 34, 35 in Fig. 2). Further improvement in already cooperative clique networks requires community-to-community connections but in a controlled way without increasing short cycles that reduce support for cooperators at the boundary of communities. This community motif both joins together agents in cohesive groups and extends an interface to other groups as well.

The social environment is far different between the clique network model and the Barabási-Albert^47^,^48^ (BA) scale-free model. BA-graph cooperation is high relative to the fully connected graph in competitive situations, modeled by the Prisoner’s Dilemma; however, in the Stag Hunt and Snowdrift games cooperation is comparatively low but can hang on and even improve modestly as communities are connected better but this same connectivity becomes a liability under competition (Fig. 4) by allowing defection to invade through the same pathways. Taken as a whole, though, cooperation improvement is lower in scale-free graphs than well mixed or fully connected graphs, but we do find that cooperation is higher on lattices than scale free, contrary to human cooperation experiments^50^ that show both lattice and scale free networks behave the same, but aligns with the theoretical literature that shows improvements in the lattice over well mixed populations^17^,^48^,^51^.

In the clique case (Fig. 5), cooperation is robust therefore adding links between communities gives additional opportunities for reciprocal action. These same paths allow defectors to invade scale-free network hubs and this cycle of invasion erodes forgiveness and trust. Acyclic structures are an asset in competitive situations, represented by the Prisoner’s Dilemma game, because they limit the spread of defection to otherwise cooperative communities; however, when cycles are absent and coordination is needed, where agents attempt to predict^52^ future moves of their partners, acyclic communities lack sufficient support at external boundaries that cycles can provide^23^, and so are unable to efficiently coordinate which reduces fitness of the global population.

We have also developed a socially adaptive rule **SOC** in which the noise is proportional to the local environment noise in payoffs and show that these results are similar to that of strong selection (*β* > 10) and this constrains the usefulness of this rule to relatively strong selection found in microbial cooperation^53^, rather than human cooperation in which the moves of players have a more significant impact than payoffs^54^.

In this work, we have studied cooperation emergence in both the BA and clique structures through subtle single-edge network changes. We are unaware of any comparable study that delves into single-edge effects, nor the effect of network motifs on cooperation emergence, which we show to be linear in our clique example (Fig. 5). We also confirm, for reactive strategies, that scale-free network cooperation is significantly reduced, as reported by^21^,^22^, and confirm the robustness of these results from weak to strong selection, including a socially adaptive rule (**SOC**).

Research that extends the present work includes investigating the size and connectivity of graph clique modules and extending the strategy space to a four-part framework that includes the Pavlov strategy, for example extending^55^ and the present work.

## Methods

**Figure 7:**
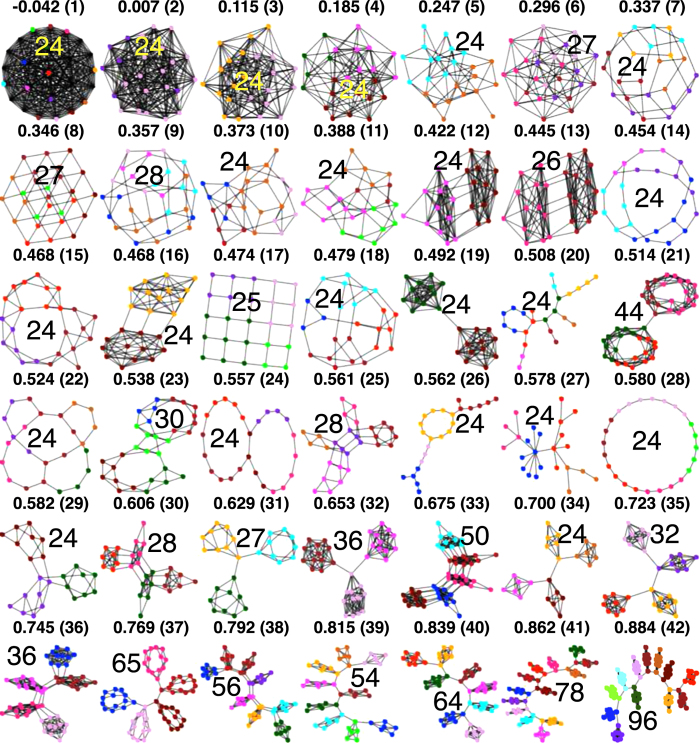
Networks. All graphs, except for the edge addition BA and clique experiments in are shown in this figure. The modularity value (*Q*^32^) is shown above each panel along with the graph index in parentheses. Individual communities or modules are indicated by color where nodes with the same color belong to the same community or module. Community detection is performed by a random walk method described by^60^. The number of nodes is shown on each graph in the figure.

We evolve our population of social dilemma game players in a network environment (see Fig. 7) where nodes represent players and links the interactions where games are played. A prior modularity study^17^ used an updating rule from^18^; however, this rule gives up the ability to adjust selection strength. In this work we apply the more conventional Fermi rule, equation (1), in order to take advantage selection adjustment. Additionally,^17^ performed synchronous updating whereas in the present work we employ asynchronous updating. Finally, we modified the *y*, *p*, *q* stochastic strategy framework, from^43^,^44^, slightly by making *y* a discrete rather than continuous variable. These changes were largely motivated by the need to decrease simulation convergence time and we found that these rules, especially asynchronous updating, decreased convergence time considerably. These changes also allowed us to confirm the modularity effect on cooperation^17^, with asynchronous updating and the Fermi rule, from weak to strong selection.


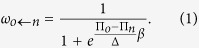


In the Fermi equation (1), 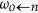 is the probability that node *o* will adopt the strategy of neighbor *n*, 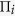, and 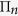 are the payoffs of *o* and neighbor *n* respectively, *β* is the evolutionary selection strength and Δ is the difference between the maximum and minimum payoff achievable in a given game. The later Δ parameter allows us to vary payoffs across a wide range of games and yet evolve the population in a common way^54^. As 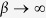 a small payoff advantage results in a high probability of switching strategies conversely when 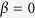 the probability of switching strategies becomes 1/2 irrespective of payoff.

Besides the fixed notion of *β* we define a dynamic value, equation (2), that adapts to the local noise of a node’s neighborhood; 1/*β* is proportional to noise^56^,^57^,^58^. The rationale for this is based on a social context where decision-making confidence is not fixed but relative to the situation or local environment that a player experiences. If I see a variety of payoffs in my neighborhood I am less confident with simply imitating the most successful since the environmental uncertainty adds doubt to the observed payoff values that may change. Conversely, I am more likely to take a risk if the environment is certain. We refer to this social rule as **SOC** in this work. In equation (2), 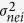 is the neighborhood variance of node *o*, and 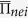 is the mean payoff of the same neighborhood.


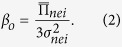


The **SOC** rule has the added advantage of reducing early imitation errors in our simulations since initial strategies 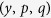 are picked from a uniform distribution for each run, a static *β* results in volatile early generations where it is common for nodes to cycle through several strategies before becoming satisfied.

The payoff 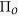, equation (3) from^17^, for each node *o* is determined by averaging the sum of its repeated game payoff over all of its nearest neighbors. The number of neighbors (degree) of node *o* is *k*_*o*_, the repeated game discount factor *w* is drawn from a uniform distribution between 0.8 and 0.9, and the number of games 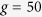.


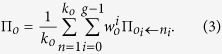


Our asynchronous updating scheme follows the coevolution method described by^46^ with the exception that we do not evolve structure but rather evolve the population to convergence before starting anew with a different network. In this scheme, one node is picked at random from the network and then one of its neighbors is chosen at random. Each of the chosen nodes (players) plays a repeated game versus their immediate neighbors and record their average payoff. Strategies of the chosen nodes update according to the Fermi function, equation (1). Updates continue to take place until convergence is achieved. We define convergence as 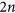 asynchronous updates with no strategy switching (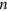 is the number of nodes in the network). If a run does not converge within 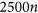 updates then the last 100 updates are averaged together; this occurs in approximately 10% of cases in the Snow Drift game but rarely in other games (the problem with Snow Drift convergence was also reported by^46^). We also averaged 1000 updates for non-converging cases and found no significant difference compared to an average of 100 updates.

All runs were executed on a 256-core, CrayXE6 computer. In total, 1024 runs were collected for each network structure, game, and *β* value in order to sample the ST payoff plane sufficiently. More samples were needed to improve the clarity of the ST-planes in Fig. 1, thus a total of 10240 samples were collected, per game, for the full, BA, and clique graphs. For the payoff matrix, similar to^17^, we adopt a simplified symmetric two-player repeated game, shown in equation (4) from^2^,^59^, where the defecting player receives *T* (temptation to defect) if their opponent cooperates but receives *P* = 0 if their opponent defects, (the punishment for mutual defection). Here we extend the PD game 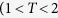 and 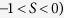 from^17^ to the Stag Hunt 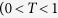 and 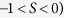, Snow Drift 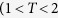 and 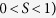, and Harmony games 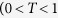 and 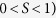. For each run, new *T* and *S* values are drawn from a uniform distribution within each respective game region.


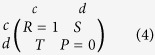


The modularity index was calculated based on community structure measurement *Q* developed by^32^, where *Q* = 1 is a maximum, or the strongest community structure. Community detection was done by a random-walk^60^ method.

## Additional Information

**How to cite this article**: Gianetto, D. A. and Heydari, B. Sparse cliques trump scale-free networks in coordination and competition. *Sci. Rep*. **6**, 21870; doi: 10.1038/srep21870 (2016).

## Supplementary Material

Supplementary Information

## Figures and Tables

**Table 1:** Summary of network effects.

Factor	HG	SH	SD	PD	Level	Description	Fig.
degree	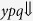	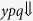	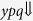	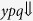	global	sparse conn. 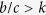 15	2
mod.	—	—	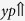	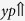	global	17, if not dense	2
long c.	—	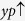	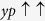	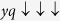	local	girth^24^, boundary^23^	4
short c.	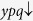	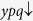	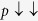	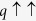	local	opposite long cycle^24^	4
motifs	—	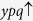	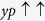	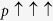	local	helps reciprocity	5

The table shows each of the key effects in this paper, 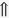 and 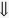 are the most significant effects, which act at the global level, local effects are shown by ↑ and ↓. Games with no clear effect are shown with a ‘–’. Multiple arrows indicate the effect (i.e., slope) relative to other games. The *y*, *p*, *q* parameters that participate in the effect are indicated beside the arrows. The short cycle effect is determined by comparing the points h (two-cycle) versus i (split cycle) in Fig. 4(A).
