# Supplementary material for: Sparse cliques trump scale-free networks in coordination and competition
Source: Sci Rep. 2016 Feb 22;6:21870. doi: 10.1038/srep21870 (PMC4761901; doi:10.1038/srep21870)
Supplement: Supplementary Information [file srep21870-s1.pdf]

Sparse cliques trump scale-free networks in coordination and competition

David A. Gianetto

Babak Heydari

Cooperation versus T for sample networks (SI1)

For pure strategies on a square lattice, [1] finds a power-law relationship between temptation to defect $T(b)$ in the PD game and cooperation level, which can be related to *two-dimensional directed percolation* according to [1], and high $T(b)$ results in cooperators disappearing from the population entirely when $T(b) > 1.6$ with $\beta = 2$. We show a different effect for stochastic reactive strategies (see figure 1) where cooperation declines with $T(b)$ but does not disappear with high $T(b)$ and the trend is more linear than exponential in nature. This result holds for the Snowdrift and Stag Hunt games. Further, q is unaffected by $T(b)$ in the PD game but declines with the other components in the SD and SH games. In the SH game, for high $T(b)$, reciprocity p and trust y do not decline as they do for the full graph (figure 1 SH panel), the decline is near linear for the lattice though the decline becomes much more steep for the full graph above $T(b) > 1/2$.

The lattice (figure 1) and clique (figure 3) structures essentially behave the same in the PD game versus $T(b)$, though cooperation on the clique graph is higher for all $T(b)$. Cooperation in the scale free graph (figure 2) is higher than the full graph in the PD but the trend versus $T(b)$ is flat.

---

[1] Szabó, G. & Hoke, C. Evolutionary prisoners dilemma game on a square lattice. *Physical Review E* **58**, 69 (1998).

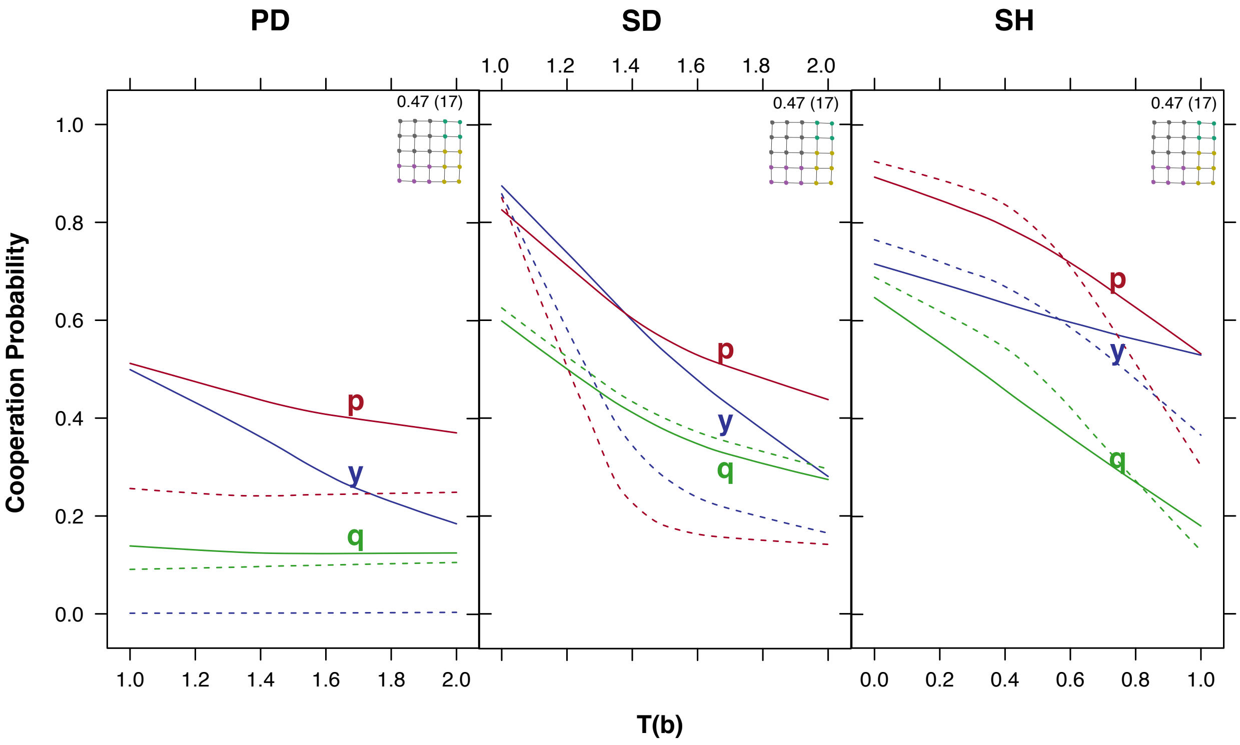

FIG. 1. Cooperation part versus $T(b)$ for a square lattice.

The figure shows the local least squares trend in cooperation for each stochastic component, for the PD, SD, and SH games. $\beta = 1$ for all games. Dashed lines are trends for the full graph.

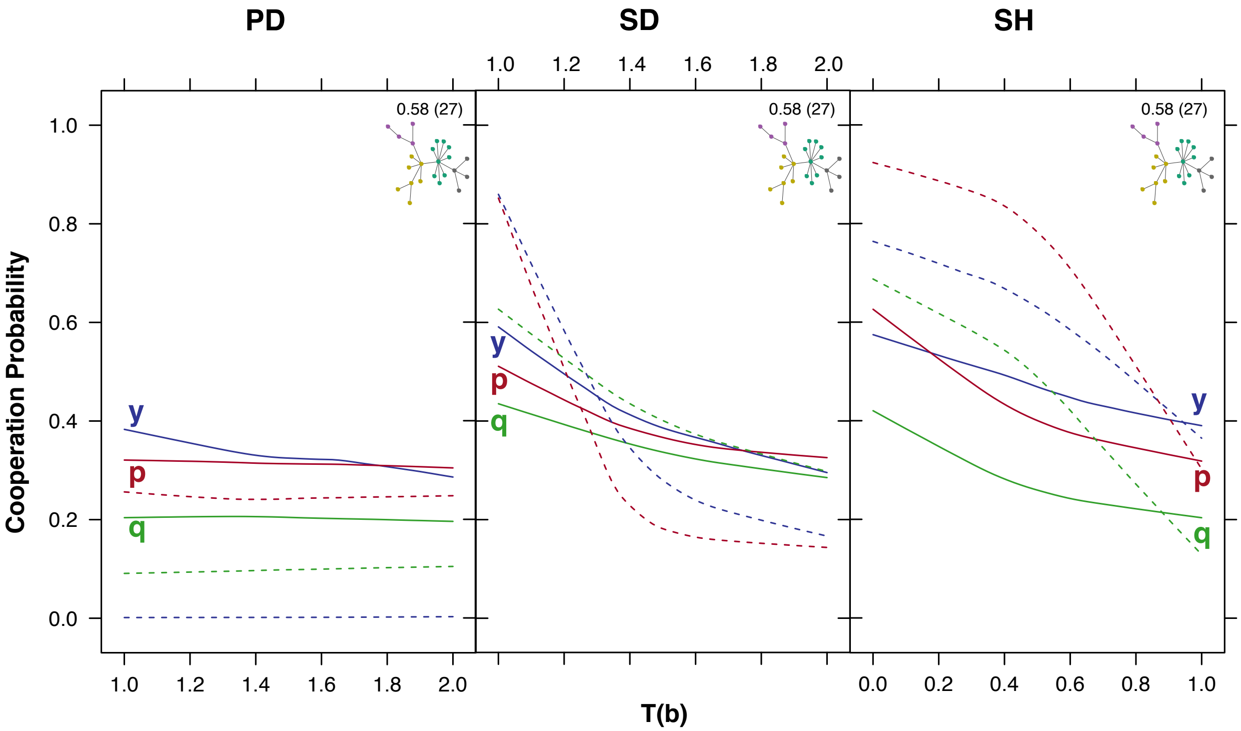

FIG. 2. Cooperation part versus $T(b)$ for a scale free graph.

The figure shows the local least squares trend in cooperation for each stochastic component, for the PD, SD, and SH games. $\beta = 1$ for all games. Dashed lines are trends for the full graph.

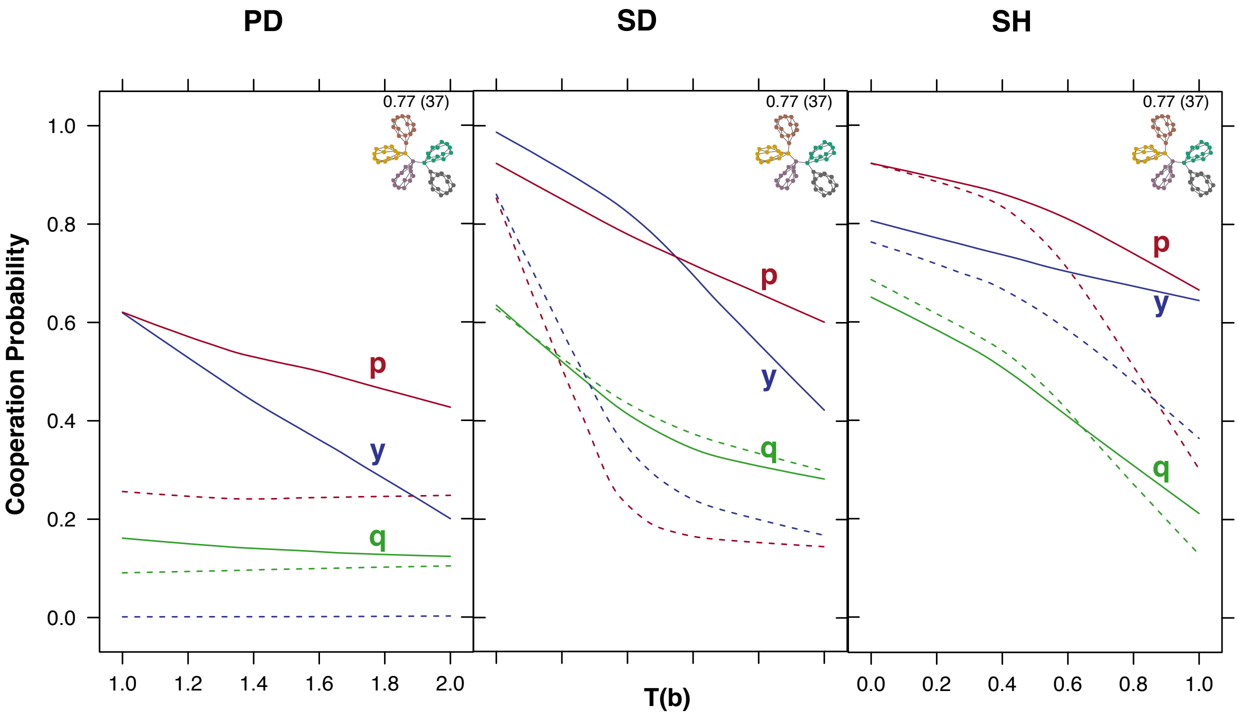

FIG. 3. Cooperation part versus $T(b)$ for a clique graph.

The figure shows the local least squares trend in cooperation for each stochastic component, for the PD, SD, and SH games. $\beta = 1$ for all games. Dashed lines are trends for the full graph.
